# Can early introduction of specialized palliative care limit intensive care, emergency and hospital admissions in patients with severe and very severe COPD? a randomized study

**DOI:** 10.1186/1472-684X-13-47

**Published:** 2014-10-21

**Authors:** Catherine Weber, Jerome Stirnemann, François R Herrmann, Sophie Pautex, Jean- Paul Janssens

**Affiliations:** 1 Department of Community Medicine, Primary Care and Emergency Medicine, Geneva University Hospitals and University of Geneva, Geneva University Hospitals 4 rue Gabrielle-Perret-Gentil, Genève 14 1211, Suisse; 2 Department of Internal Medicine, Geriatrics and Rehabilitation, Geneva University Hospitals and University of Geneva, Geneva University Hospitals 4, rue Gabrielle-Perret-Gentil, Genève 14 1211, Suisse; 3 Department of Medical Specialties, Division of pulmonary diseases, Geneva University Hospitals and University of Geneva, Geneva University Hospitals 4, rue Gabrielle-Perret-Gentil, Genève 14 1211, Suisse

## Abstract

**Background:**

COPD is a progressive lung disorder with rates of mortality between 36–50%, within 2 years after admission for an acute exacerbation. While treatment with inhaled bronchodilators and steroids may partially relieve symptoms and oxygen therapy may prolong life, for many patients the course of the disease is one of inexorable decline. Very few palliative care intervention studies are available for this population. This trial seeks to determine the effectiveness of the introduction of specialized palliative care on hospital, intensive care unit and emergency admissions of patients with severe and very severe COPD.

**Methods/Design:**

The study is a three year single centre, randomized controlled trial using a 2 arms parallel groups design conducted in a tertiary center (University Hospitals; Geneva). For the intervention group, an early palliative care consultation is added to standard care; the control group benefits from standard care only. Patients with COPD defined according to GOLD criteria with a stage III or IV disease and/or long term treatment with domiciliary oxygen and/or home mechanical ventilation and/or one or more hospital admissions in the previous year for an acute exacerbation are eligible to participate. Allocation concealment is achieved using randomisation by sealed envelopes. Our sample size of 90 patients/group gives the study a 80% power to detect a 20% decrease in intensive care unit and emergency admissions – the primary endpoint. All data regarding participants will be analysed by a researcher blinded to treatment allocation, according to the "Intention to treat" principle.

**Discussion:**

Given the trends toward aggressive and costly care near end-of-life among patients with COPD, a timely introduction of palliative care may limit unnecessary and burdensome personal and societal costs, and invasive approaches. The results of this study may provide directions for future palliative care interventions in this particular population.

**Trial registration:**

This trial has been registered at clinicaltrials.gov under NCT02223780

## Background

Chronic obstructive pulmonary disease (COPD) is the fourth leading cause of mortality and the 12th leading disability worldwide [1,2]. It is a progressive lung disorder with mortality rates between 36–50%, within 2 years after admission for an acute exacerbation [3]. One out of eight (130,000) hospital admissions per year in the UK are related to COPD with approximately 30% of patients admitted with COPD for the first time being re-admitted within 3 months [4].

While treatment with inhaled bronchodilators and steroids may partially relieve symptoms and Noninvasive Positive Pressure Ventilation (NIV) and long term oxygen therapy (LTOT) may prolong life, for many patients the course of the disease is one of inexorable decline with a prolonged period of disabling dyspnea and increasingly frequent hospital admissions reflecting deteriorating lung function and usually presaging a premature death [5]. It has long been recognized that, in this group of patients, both quality of life and survival are poor. For instance, in the Nocturnal Oxygen Therapy trial of LTOT, disturbances in emotional and social functioning were common and there was a marked impairment in activities of daily living [6].

Despite these issues, the needs of these patients are typically poorly addressed, and many patients have limited access to specialists in palliative care services [7-10]. Furthermore many of these patients are housebound and they and their caregivers have poor access to health services. No randomised early palliative care intervention studies are available for this particular population, although it has been shown that it is feasible to provide palliative care interventions in this population [11-15] A study conducted in the UK found that patients with COPD are much less likely to die at home and to receive palliative care services than patients with lung cancer [7]. Additional studies have also documented the poor quality of palliative care and significant burden of symptoms among patients with COPD [7,8,16-19]. In a retrospective cohort study conducted in Canada, of 1098 patients who died in 2004, very few patients who died with COPD used the palliative care services that were available in acute care hospitals (5.1%) or home care settings (2.8%) compared to those with lung cancer (acute care 47.6%; home care 37.4%) [20].

Although anxiety and depression are common in COPD, they are not well recognized or treated [21]. In a recent study, only a third of patients with COPD and clinically significant depression or anxiety were being treated and only half of those with severe depression or anxiety were being treated [22]. Antidepressants can significantly improve mood among patients with COPD and depression [23].

One reason these patients may receive poor-quality palliative care is that patient–physician communication about end-of-life care often occurs late in the illness [24,25]. Healthcare for these patients is often initiated in response to acute exacerbations rather than being initiated proactively based on a previously developed plan for managing their disease [26].

Studies have shown that only a minority of patients with moderate-to-severe COPD has discussed treatment preferences and end-of-life care issues with their physicians and most believe that their physicians do not know their preferences for end-of-life care [24,25,27]. A survey of 214 general practitioners in the UK found that, although 82% felt that general practitioners should discuss prognosis with COPD patients, only 41% reported "often" or "always" discussing prognosis with these patients [28]. Furthermore, two thirds of the physicians who reported infrequently discussing end-of-life care reported feeling inadequately prepared to have such discussions.

The "SUPPORT" study (study to understand prognosis and preferences for outcomes and treatment) that/which enrolled seriously ill hospitalized patients, including COPD, demonstrated that COPD compared to patients with lung cancer, were much more likely to die in the intensive care unit, on mechanical ventilation and with dyspnea [29]. These differences occurred despite most patients with COPD preferred treatment focused on comfort rather than on prolonging life. In fact, SUPPORT found that patients with lung cancer and patients with COPD were equally likely to prefer not to be intubated and not to receive cardiopulmonary resuscitation (CPR), yet patients with COPD were much more likely to receive these therapies [29]. A study of patients with COPD or lung cancer in the US Veterans Affairs Health System also found that patients with COPD were much more likely to be admitted to an ICU, and have greater lengths of stay in the ICU during their terminal hospitalization, than patients with lung cancer [30].

Most patients with a life-limiting illness prefer to discuss their preferences for end-of-life care relatively early in the course of their illness, as these discussions are less stressful when the patient is feeling relatively well [31]. One qualitative study compared patients with severe COPD to patients with metastatic cancer or advanced AIDS, showed that patients with COPD were more likely to express concern about the lack of information that they received about their disease. They wanted more information in the following five specific areas: diagnosis and disease process, treatment, prognosis, what dying might be like, and advance care planning [32].

A recent randomized study conducted in the United States in an outpatient clinic demonstrated that an intervention using patient specific feedback about preferences for discussing end-of-life care improved the occurrence and the quality of communication between 376 patients with COPD and their clinicians. Patients in the intervention group reported nearly a threefold higher rate of end-of-life discussions about end-of-life care (30% vs. 11%; P < 0.01) [33].

Advance directives may be especially useful among patients with COPD because of their likely trajectory of illness, with unpredictable exacerbations that may render patients suddenly critically ill. Some patients with COPD have strong feelings about the situations in which they would want to forego CPR or, particularly relevant for COPD, mechanical ventilation for acute respiratory failure. Patients’ prior experiences with life support, or with relatives or friends who have required life support, can be important facilitators for patient–physician communication about treatment preferences and end-of-life care [34]. The period of hospitalization may not be an appropriate time to initiate advance directives, but the opportunity should be taken after discharge from hospital [35].

Because of the paucity of palliative care intervention studies available for this particular population, we designed a study to evaluate the effect of introducing palliative care for patients with severe and very severe COPD.

Our hypothesis is that the early introduction of specialized palliative care could decrease the length and number of admissions to intensive care units as well as hospital and emergency admissions of patients with severe and very severe COPD.

The primary objective of the study is to assess the effectiveness of the introduction of early specialized palliative care on hospital, intensive care unit and emergency admissions of patients with severe and very severe COPD.

The secondary objectives are to assess impact of early palliative care on:

1. mood and anxiety of patients with severe and very severe COPD

2. health-related quality of life of patients with severe and very severe COPD.

3. pattern of antibiotics used during the last month of life

4. advance care planning and end-of-life decision-making

## Methods

### Design of the study

This randomised controlled trial will used 2 arms parallel groups design. The intervention group will benefit from an early specialized palliative care intervention in addition to standard care. The control group will benefit from standard care. Consenting participants will be randomly allocated to one of the groups after baseline assessment. Patients will continue to have the same access to specialized medical services as available to them prior to entry into the trial.

### Setting

The study will be conducted in the largest tertiary university hospital in Switzerland. Potentially eligible patients will be identified during their attendance at the ambulatory consultation of the Division of Pulmonary Disease and/or during their hospitalization in the Division of General Internal Medicine at Geneva University Hospital.

The Division of Pulmonary Disease provides follow-up care for LTOT and/or NIV for an important proportion of COPD in Geneva. Furthermore it is the only public hospital in the Geneva area and most of the patients with COPD are admitted to the Division of General Internal Medicine if they present an acute exacerbation.

The area has a palliative care network with a community palliative care unit, involved in the project. Very few patients with COPD are addressed to this unit.

### Inclusion and exclusion criteria

The inclusion criteria are:

COPD defined according to GOLD criteria (FEV_1_/FVC <70%) stage III or IV (FEV_1_ < 50% predicted) [36]

– and/or long term treatment with either domiciliary oxygen [37] or home mechanical ventilation [5]

– and/or one or more hospital admissions in the previous year for an acute exacerbation

The exclusion criteria are:

– Patients in their last days of life ( patient bedbound and/or semi comatose and/or take only fluid and/or no longer able to take oral drugs)

– Patients with cognitive impairment: Mini Mental status Examination ≤23 at the day of inclusion of patients [38]

– Patients with active cancer

### Recruitment procedure

Potential participants will receive information from our research nurse and will be advised as to what is involved if participating in the trial. Potential trial participants will be informed that they have a 50% chance of being allocated to the intervention group and a 50% chance to receive standard care. Reasons for declining to participate will be recorded as: not interested, too busy, don’t believe in it, declined randomisation, other. Other reasons for leaving the study prior to randomisation will be noted.

The study nurse will arrange an appointment for the baseline assessment with the patient. This first appointment will take place in the patient’s home or at the hospital, according to patient’s choice. At this appointment, written informed consent for participation in the study will be obtained from participants.

### Randomisation-allocation concealment

After giving written informed consent and filling in baseline measures, the patients will be randomly assigned to the "early palliative care" group or the "standard care only" group using sealed envelopes prepared using a computer program by FRH co investigator not involved in the inclusion of the patients, in a 1:1 ratio without stratification and with randomized block sizes ranging from 4 to 6.

### Ethics approval

The Ethics Commission of the University Hospital Geneva approval has been obtained prior to recruitment. All patients will provide written informed consent. Recruitment started in September 2013.

### Intervention

The theoretical basis of the intervention was established by reviewing the published literature on the impact of the early introduction of palliative care in patients with severe diseases [7,8,12,14,16-18,22,24,32-34,39-49]. Information gathered in this phase has been used to develop the optimal intervention by a multidisciplinary group and a study design adapted to our regional health system. Researchers involved in the recruitment of the patients are blinded to the details of the intervention. Our team also established contacts with experts who had already developed specialized palliative care interventions in this type of population.

Patients assigned to the early palliative care group will meet a nurse attached to the community ambulatory palliative care unit of the HUG (USPC) within three weeks of inclusion and at least monthly thereafter during one year after inclusion. This nurse has a long experience in palliative care. The first consultation will take place at the patient’s home, unless the patient prefers to have the consultation at the pulmonary division. It will take approximately 1 h ½ to assess and discuss the different items. The subsequent monthly consultations will last approximately 30 minutes according to the needs of the patients. A consultation with a specialized palliative care physician or another specialist (psychiatrist, pulmonologist….) will be organized if intensity of pain, dyspnea, mood, anxiety and appetite disturbances are >4/10 on visual analogic scales (VAS) and both the patient and his/her physician agree. The individualised palliative care assessment and treatment plan will be forwarded in writing to the physician in charge of the patient within 24 hours of the consultation. These consultations will be at charge of the insurance of the patient. Details of the palliative consultation are described in Table 1. We decided to establish a set of core interventions, but not to schedule these different items to respect the rhythm and needs of the patients. At the end of each consultation, the items discussed during the consultations will be collected. Participants who refuse to attend one or more palliative care interventions will be contacted by phone by the palliative care nurse to understand their reasons. If the patient, agrees a new appointment will be fixed.

**Table 1:** Description of the palliative care intervention

1.	**Symptom management**
	- Assessment of symptoms with the Edmonton Symptom Assessment Scale
	- If intensity of pain, dyspnea, mood, anxiety and appetite >4 and patient agrees: patients will be referred to home based specialist palliative care team or another specialist (dieticians, pulmonologist, psychiatrist…)
	- Symptom management will follow the palliative care guidelines of the University Hospital of Geneva.
	- Non pharmacological interventions such as relaxation will be promoted according to Booth [42]
2.	**Understanding of illness and coping with the disease:**
	**-** History of disease made with the patient
	**-** Experience of patient with breathlessness
	**-** Information about dyspnea and the disease (discussion and booklet)
	**-** Information about morphine if needed (discussion and booklet)
3.	**Anticipation**
	**-** Values of the patients (what is important for you)
	**-** Patient preferences for end-of-life care: hospitalization f.ex
	**-** Information about advance directives, nomination of surrogate decision maker (booklet and discussion)
4.	**Relatives support**
	**-** Identification of the most important relative
	**-** Assessment of their needs
	**-** Provision of available resources
5.	**Social support**
	**-** Assessment of needs (financial, administrative.)
	**-** Provision of available resources
6.	**Spiritual support**
	**-** Assessment of needs
	**-** Provision of avalables ressources
7.	**Coordination of the health professionals**
	**-** Listing of the health professionals involved in the situation
	**-** Discussion with them of goals of care

### Control group

Patients randomized to the control group, i.e. who receive standard care alone, will not meet with the palliative support team unless the patient himself, his family or his treating physician request a meeting. These patients will remain in the control group, as this is already part of present practice.

Patients who present acute exacerbations will be treated by the participant’s GP or pulmonologist or by the emergency division according to usual practices and recommendations of our institution.

### Outcomes measures

Regarding the primary outcome, Hospital, ICU and emergency admissions will be collected in both groups form the medical records once a month. Length of stay in the different divisions, type of divisions (i.e.: internal medicine, rehabilitation) and reasons for admissions will be collected.

Regarding the secondary outcomes of the study:

– Mood and anxiety of patients will be measured with the self-assessment instrument: the Hospital Anxiety and Depression Scale (HADS) [50,51]

– Quality of life will be measured with the SF-36 short form: a self-assessment scale validated in this population, and the COPD Assessment Test (CAT): a short, simple instrument for quantifying the symptom burden of COPD in routine practice

– Use of antibiotics during the last three months will be recorded

– Completion of advance directives, documented preferences for resuscitation, or nomination of surrogate decision maker will also be recorded

The secondary outcomes will be collected at baseline and at weeks 12, 24, 36 and 48.

### Data collection

Baseline data will be collected in each patient’s home by the study nurse. The timing of interventions and data collection has been designed to not be too burdensome in this population with a severe disease [15].

The baseline characteristics of the participants that will be measured are:

– Socio-demographic items: age, sex, marital status, education, children, living condition

– Smoking habits, weight, height, co-morbidities measured by the CIRS [52], activities of daily living [53], artificial nutrition (enteral, parenteral), prescribed treatment

– health professionals who participated to their care over the past three months (ie: nurses , dietician, occupational therapist)

– Additional equipment such as electric bed, wheelchair

– Characteristics and management of COPD: pulmonary function tests, oxygen saturation, use of oxygenotherapy, use of NIV, pharmacological treatment, numbers of acute exacerbations, admissions to emergency room, intensive care unit and hospital over the past three months Location and date of death will be collected from the medical records

Most of these data will be collected during the collection of primary and secondary outcomes (at weeks 12, 24, 36 and 48).

Data will be collected in computer files and will be rendered anonymous, without any reference to initials. Only the principle investigator will have the key to the correspondence between patient reference number and identity. The CRF (case report form) will be collected using SecuTrial software with the support of the Clinical Research Center, Geneva University Hospitals (Prof. Jules Desmeules). A clinical research fellow, allocated for this project will adapt the data base and enter the data in the SecuTrial software.

### Statistics

To compare baseline characteristics and study outcomes of the patients between groups, Fishers’ exact tests, chi-square tests and the independent-samples t-test or the non-parametric equivalent Mann–Whitney u test will be used.

### Power calculation

One hundred-thirteen patients are currently followed by the Division of Pulmonary Disease in the University Hospital Geneva because of treatment with NIV and/or LTOT. We know that between one third and one half of these patients are hospitalized at least once during the year. Furthermore approximately 200 COPD patients per year are admitted to the Emergency Division of our hospital. Even if only a third of these patients are included in the study, this would give us sufficient statistical power to validly analyze our primary outcomes. Regarding the primary outcome, we calculated that a sample size of 80 patients per group would be adequate with an 80% power to detect a difference in hospital admissions of 20% between both groups at an α significance level (2-sided test) of 0.05.

With regard to missing data, we will use the conservative approach of carrying the last observation forward for all missing data in the intention-to-treat analyses. Poisson or binomial negative regression models will be used to compare count data between the groups while adjusting for potential confounder. The trend in the evolution of the different scores will be assessed using mixed linear regression models.

## Discussion

Given the trends toward aggressive and costly care near the end-of-life among patients with COPD, timely introduction of palliative care may decrease unnecessary and burdensome personal and societal costs. The results of this study may provide some directions for future palliative care interventions in this particular population. These interventions may also provide a basis for training physicians and health professionals to improve patient–clinician communication about end-of-life care for patients with COPD, and stimulate the development of a specialized palliative care consultation for this population.

This is one of the first randomized trials to measure the effectiveness of early palliative intervention on hospital, intensive care unit and emergency admissions of patients.

One limit of the study is that the nature of the intervention makes it difficult to double-blind comparisons between both groups. However every attempt will be made to ensure that the researchers that include and follow the patients are blinded to the palliative care intervention.

The study is supported by the Swiss National Science Foundation grant # 406740_145086/1( http://www.snf.ch).

## Competing interests

The authors declare that they have no competing interests.

## Authors’ contributions

CW, FH, JPJ have contributed to overall concept and design. CW, SP and JPJ designed and co-ordinated the final content of the intervention. CW, JS and JPJ have organised acquisition of data and analysis of data. CW, SP, JS, FH and JPJ were involved in drafting the manuscript. Each author has participated sufficiently in the work to take public responsibility for appropriate portions of the content. JPJ has been responsible for acquiring funding and has supervised the research. All authors have read and approved the final version of the manuscript.

## Pre-publication history

The pre-publication history for this paper can be accessed here:

http://www.biomedcentral.com/1472-684X/13/47/prepub
